# Paired [^18^F]FDOPA and [^18^F]FDG PET Shows Complementary Prognostic Associations at Recurrence in IDH-Wildtype Glioblastoma

**DOI:** 10.3390/cancers18203333

**Published:** 2026-10-15

**Authors:** Julien Todeschi, Julien Salvadori, Oreste Allegrini, Hélène Cebula, Caroline Bund, Izzie-Jacques Namer

**Affiliations:** 1 Service de Neurochirurgie, Hôpitaux Universitaires de Strasbourg (HUS), 67200 Strasbourg, France; julien.todeschi@chru-strasbourg.fr (J.T.); helene.cebula@chru-strasbourg.fr (H.C.); 2 ICube, Université de Strasbourg/CNRS (UMR 7357), 67085 Strasbourg, France; c.bund@institut-strauss.fr; 3 Service de Médecine Nucléaire et d’Imagerie Moléculaire, Institut Strauss, 67200 Strasbourg, France; j.salvadori@institut-strauss.fr (J.S.); o.allegrini@institut-strauss.fr (O.A.)

**Keywords:** glioblastoma, recurrence, [^18^F]FDOPA PET, [^18^F]FDG PET, metabolic tumor volume, prognosis

## Abstract

Background/Objectives: Prognostic PET biomarkers in glioblastoma remain mostly as a single tracer and poorly characterized at recurrence. We investigated whether paired [^18^F]FDOPA/[^18^F]FDG PET captures complementary metabolic information associated with survival in IDH-wildtype glioblastoma. Methods: Sixty-five consecutive patients underwent paired [^18^F]FDOPA/[^18^F]FDG PET/MR at baseline and/or recurrence. Survival was analyzed from baseline PET/MR (OS1, *n* = 36) and recurrence PET/MR (OS2, *n* = 55); longitudinal analyses used the paired subgroup (*n* = 26). FDOPA and FDG tumor masks were independently segmented within a FLAIR-derived tumor box, and metrics were extracted from tracer-specific masks, their intersection, and their union. Metrics significant in Kaplan–Meier or univariable Cox analyses were tested in adjusted Cox models. Benjamini–Hochberg false discovery rate correction was used as a sensitivity analysis for the continuous Cox screening tests. Results: In exploratory screening, prognostic associations were sparse at baseline and more prominent at recurrence. At OS2, three metrics remained independently associated with survival after adjustment: the FDOPA/FDG union-domain SUV_mean_^union^ ratio (HR, 1.50; *p* = 0.0168), MTV^inter^ (HR, 1.63; *p* = 0.0154), and MTV^DOPA^ (HR, 1.58; *p* = 0.0191). The SUV_mean_^union^ ratio and MTV^DOPA^ showed limited correlation, suggesting complementary information. Their integration into IR-MTV^DOPA^, defined as FDOPA/FDG SUV_mean_^union^ ratio × log(MTV^DOPA^), showed higher apparent OS2 discrimination (HR, 1.73; log-rank *p* = 0.0001; C-index, 0.730; bootstrap .632-corrected C-index, 0.684; 12-month AUC, 0.863). Conclusions: In this exploratory single-center cohort, paired [^18^F]FDOPA/[^18^F]FDG PET showed prognostic associations at recurrence. Inter-tracer intensity balance and metabolic tumor burden provided partially complementary survival information, and their exploratory integration showed higher apparent discrimination. External validation is required before clinical implementation.

## Simple Summary

Patients with glioblastoma almost invariably experience recurrence, but prognosis after recurrence varies substantially. PET imaging can assess tumor metabolism, although most studies use a single tracer. We studied 65 patients with IDH-wildtype glioblastoma who underwent paired [^18^F]FDOPA and [^18^F]FDG PET/MRI before treatment and/or at recurrence. By analyzing both tracers within the same tumor regions, we found that prognostic information was limited before treatment but became more informative at recurrence. Two complementary features were associated with survival after recurrence: the balance between FDOPA and FDG uptake and the volume of metabolically active tumor. An exploratory composite integrating these features showed higher apparent risk discrimination than either feature alone. These findings support paired-tracer PET as a potential framework for characterizing metabolic heterogeneity at glioblastoma recurrence, but external validation is required before clinical use.

## 1. Introduction

At glioblastoma recurrence, clinicians must decide whether to pursue repeat surgery, additional radiotherapy, or systemic treatment in a setting where no retreatment strategy has shown a uniform survival benefit across patients [1]. Despite standard upfront management combining maximal safe resection, radiotherapy, and temozolomide, recurrence remains almost universal and survival after relapse is limited and heterogeneous [2,3]. Reliable recurrence-specific prognostic imaging biomarkers are therefore needed to better stratify patients and support retreatment decisions.

MRI remains essential for follow-up, but post-treatment enhancement and nonenhancing abnormalities do not always reliably distinguish viable tumor from treatment-related change [4]. PET can complement MRI by providing metabolic information beyond morphology alone [5]. Current EANM/EANO/RANO/SNMMI recommendations support the use of amino-acid PET and [^18^F]FDG PET in glioma imaging across diagnosis, treatment planning, recurrence assessment, and response evaluation [6,7]. Amino-acid PET, including [^18^F]FDOPA, is particularly useful for tumor detection, delineation, and recurrence assessment because of its higher tumor-to-background contrast, whereas [^18^F]FDG PET remains widely available and can capture glucose-related tumor metabolism despite high physiologic cortical uptake [6,7]. Recent multicenter data also support FDOPA PET for distinguishing recurrent high-grade glioma from treatment-related changes [8].

Both [^18^F]FDOPA and [^18^F]FET are recognized amino-acid PET tracers in these recommendations. In our center, [^18^F]FDOPA was the amino-acid tracer routinely available throughout the study period and was therefore used for this retrospective cohort; this choice reflects local clinical practice rather than an assumption of superiority over FET.

Several PET-derived metrics have been associated with the outcome in glioma. FDOPA-derived parameters, including metabolic tumor volume, have shown diagnostic and prognostic associations in recurrent glioma, although the evidence remains heterogeneous and mostly as a single tracer [9,10,11,12]. FDG-derived uptake parameters can also carry prognostic information in glioma, particularly in recurrent high-grade glioma, but FDG is less suited for tumor delineation because of high normal-brain uptake [6,13,14]. More broadly, PET response frameworks increasingly emphasize measurable metabolic burden, underscoring the clinical relevance of volumetric PET metrics while also highlighting their dependence on tracer-specific delineation strategies [15,16]. Histologic validation has shown that FDOPA uptake may identify a tumor beyond contrast-enhancing MRI abnormalities [17]. FDOPA uptake has also been related to proliferative activity in treatment-naive gliomas [18]. More recently, PET-derived biological tumor volume has been associated with survival in recurrent high-grade glioma in a multiparametric PET/MRI cohort [19].

However, most available studies either analyze single-tracer metrics or compare tracers rather than integrating spatially aligned paired-tracer information within the same patient. Whether recurrence prognosis is primarily driven by inter-tracer intensity balance, metabolic tumor burden, or their integration remains unclear. Paired [^18^F]FDOPA and [^18^F]FDG PET/MR examinations offer the opportunity to characterize amino-acid-related and glucose-related tumor metabolism within a common spatial framework. In this study, we analyzed longitudinal paired-tracer PET changes between baseline and recurrence, screened PET metrics for association with overall survival from baseline PET (OS1) and from recurrence PET (OS2), assessed the complementarity of recurrence-associated metrics, and explored whether integrating inter-tracer intensity balance with metabolic tumor burden could improve risk discrimination.

## 2. Materials and Methods

This single-center retrospective observational study included consecutive adults (≥18 years) with histopathologically confirmed IDH-wildtype glioblastoma who underwent paired [^18^F]FDOPA/[^18^F]FDG PET/MR assessments as part of routine care, at baseline (PET1), at suspected recurrence (PET2), or at both time points. PET1 was a true pretreatment baseline examination performed before any tumor-directed treatment, including surgery, radiotherapy, or chemotherapy; for patients contributing PET1, imaging therefore preceded the diagnostic surgical procedure that subsequently established the histopathological diagnosis. PET2 was performed when recurrence was suspected on MRI and before any recurrence-directed treatment. All PET2 examinations included in the survival analyses were subsequently confirmed as recurrence based on concordant MRI and PET findings, and none was ultimately reclassified as pseudoprogression or radiation necrosis. Because PET contributed to routine clinical recurrence assessment, recurrence confirmation was not independent of the imaging under study. At each time point, [^18^F]FDOPA and [^18^F]FDG PET/MR were performed as separate examinations. The inter-scan interval was 2 days [IQR, 2–4] overall (range, 1–27 days) and 2 days [IQR, 2–2] at PET2 (range, 1–10 days); no prespecified maximum interval was imposed in this retrospective routine-care cohort.

**Figure 1:**
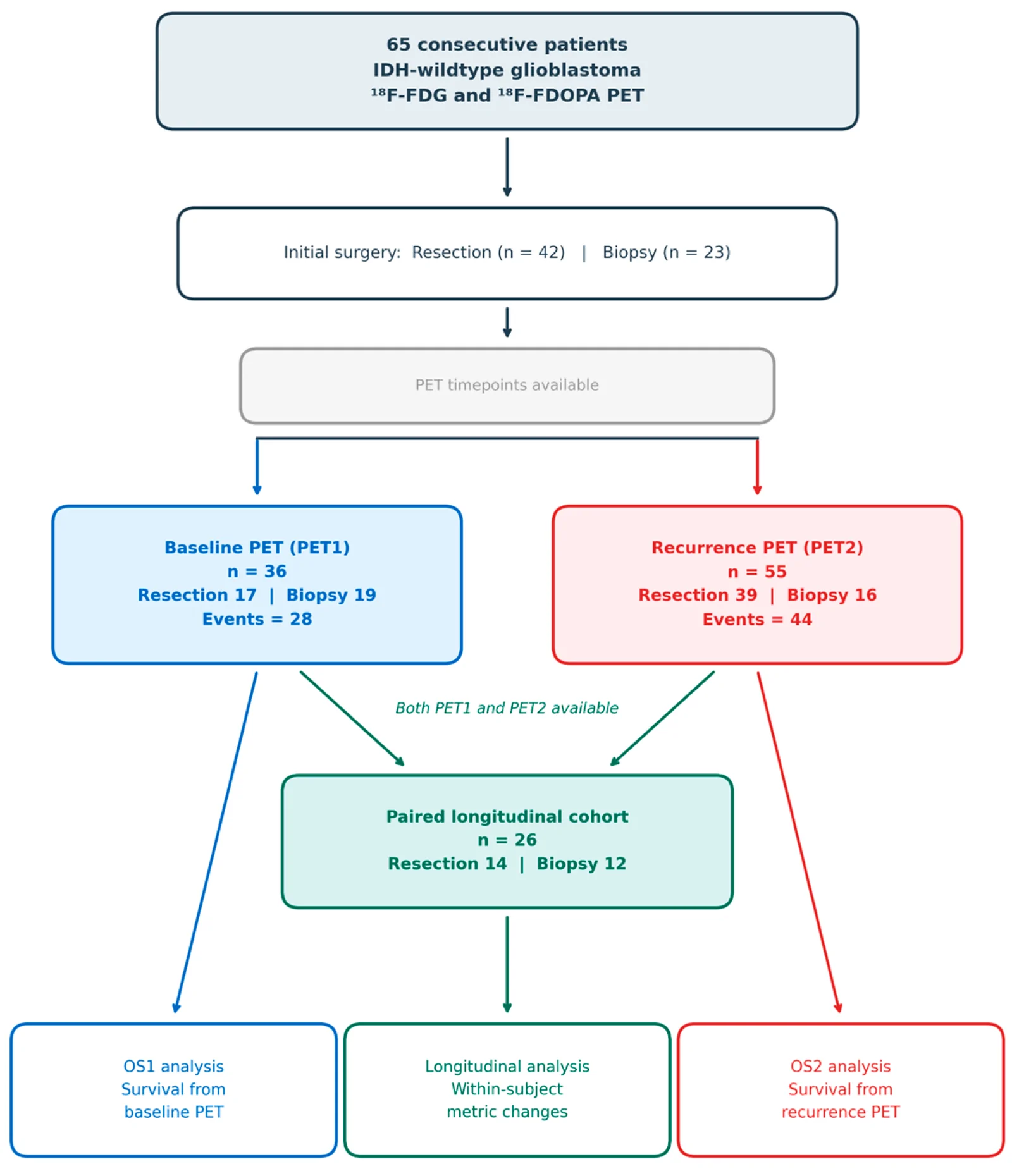
Study flowchart and analytic subsets. Flowchart of the study population and analysis subsets. The cohort included 65 consecutive patients with IDH-wildtype glioblastoma who underwent paired [^18^F]FDOPA/[^18^F]FDG PET. Subsets are shown for OS1, OS2, and paired longitudinal analyses.

The study cohort comprised 65 patients. Of these, 36 contributed to the PET1 cohort, 55 contributed to the PET2 cohort, and 26 had both PET1 and PET2 available and formed the paired longitudinal subgroup (initial resection, *n* = 14; biopsy, *n* = 12). The overall study design, available paired PET time points, and analysis subsets are summarized in Figure 1.

### 2.1. Ethics

Ethical approval was obtained from the Ethics Committee of Institut Strauss, Strasbourg, France (IRB-2025-14; approved 16 December 2025). Given the retrospective design and use of anonymized clinical and imaging data, the requirement for written informed consent was waived. Procedures complied with the Declaration of Helsinki and applicable regulations.

### 2.2. Imaging Protocol

All examinations were performed on a hybrid SIGNA 3T PET/MR system (GE Healthcare, Waukesha, WI, USA). The chemical structures of [^18^F]FDG and [^18^F]FDOPA are shown in Figure S1. For [^18^F]FDG, patients fasted for at least 6 h and preinjection blood glucose had to be below 1.3 g/L. For [^18^F]FDOPA, patients fasted for at least 4 h. For both tracers, 2 MBq/kg (0.054 mCi/kg) were injected intravenously, and a 15 min static brain PET acquisition started 30 min after injection. PET was performed simultaneously with a standardized brain MRI protocol. PET data were reconstructed with OSEM time-of-flight with standard corrections including MR-based attenuation correction using a ZTE method [20,21], SSS scatter correction, and singles-based randoms estimation [22]. All quantitative analyses used body-weight-normalized SUV images.

### 2.3. Image Processing

Image analysis followed a standardized and automated multimodal workflow. For each paired [^18^F]FDOPA/[^18^F]FDG PET/MR examination, the T1-weighted MRI acquired during the FDG examination was rigidly registered to the corresponding FDOPA examination, and the resulting transformation was applied to the associated FDG images. All images and derived masks were then resampled to a common 1 mm isotropic voxel grid.

A whole-tumor FLAIR mask, defined as the FLAIR-hyperintense lesion volume including the tumor core and peritumoral edema, was obtained using a dedicated deep-learning segmentation model trained on the BraTS reference dataset, followed by a visual review and manual correction when required [23]. Whole-tumor (WT) volume was derived from this noneroded FLAIR mask. A fixed 2 mm isotropic erosion was then applied to generate a reduced FLAIR-based mask, hereafter referred to as the FLAIR box, which was used as the search space for PET-based tumor segmentation. This step was intended to reduce boundary and partial-volume effects related to the lower spatial resolution of PET and to limit contamination from adjacent physiologic uptake.

Tumor segmentation on [^18^F]FDOPA and [^18^F]FDG PET images was then performed independently within the FLAIR box using automated thresholding based on Otsu’s method. The same data-driven segmentation strategy was deliberately applied to both tracers to preserve methodological symmetry for subsequent union, intersection, and inter-tracer ratio analyses. Restricting Otsu thresholding to the 2 mm-eroded FLAIR search space further limited inclusion of adjacent physiologic cortical uptake. This symmetric, observer-independent approach was selected a priori to enable spatial comparison of the two tracers within a common anatomical search space. A tracer-specific fixed threshold was not used as the primary paired approach because no single fixed threshold is established for both FDOPA and FDG; applying the same data-driven rule within an identical anatomical search space avoided introducing tracer-dependent segmentation rules into spatial comparisons.

As a FDOPA-specific segmentation sensitivity analysis, an additional metabolic volume was delineated using a fixed threshold of 1.6 × mean contralateral gray-plus-white-matter background activity within the same 2 mm-eroded FLAIR search space. Because five PET2 examinations yielded no measurable volume with this fixed threshold, the natural-log transformation used for the primary volume-based Cox models could not be applied consistently. This sensitivity analysis was therefore restricted to a median-dichotomized Kaplan–Meier comparison; examinations with zero measurable volume were assigned to the low-volume group.

Anatomical parcellation of T1-weighted MRI using OpenMAP-T1 (https://github.com/OishiLab/OpenMAP-T1, accessed on 11 October 2026) was used to identify contralateral gray and white matter, which served as tracer-specific reference regions for tumor-to-background ratio (TBR) calculation [24].

### 2.4. Quantitative Metrics

PET metrics were derived across 3 spatial domains designed to capture complementary aspects of tumor extent and tracer distribution: the union of the FDOPA- and FDG-defined PET tumor masks, their intersection, and the corresponding tracer-specific PET masks.

**Figure 2:**
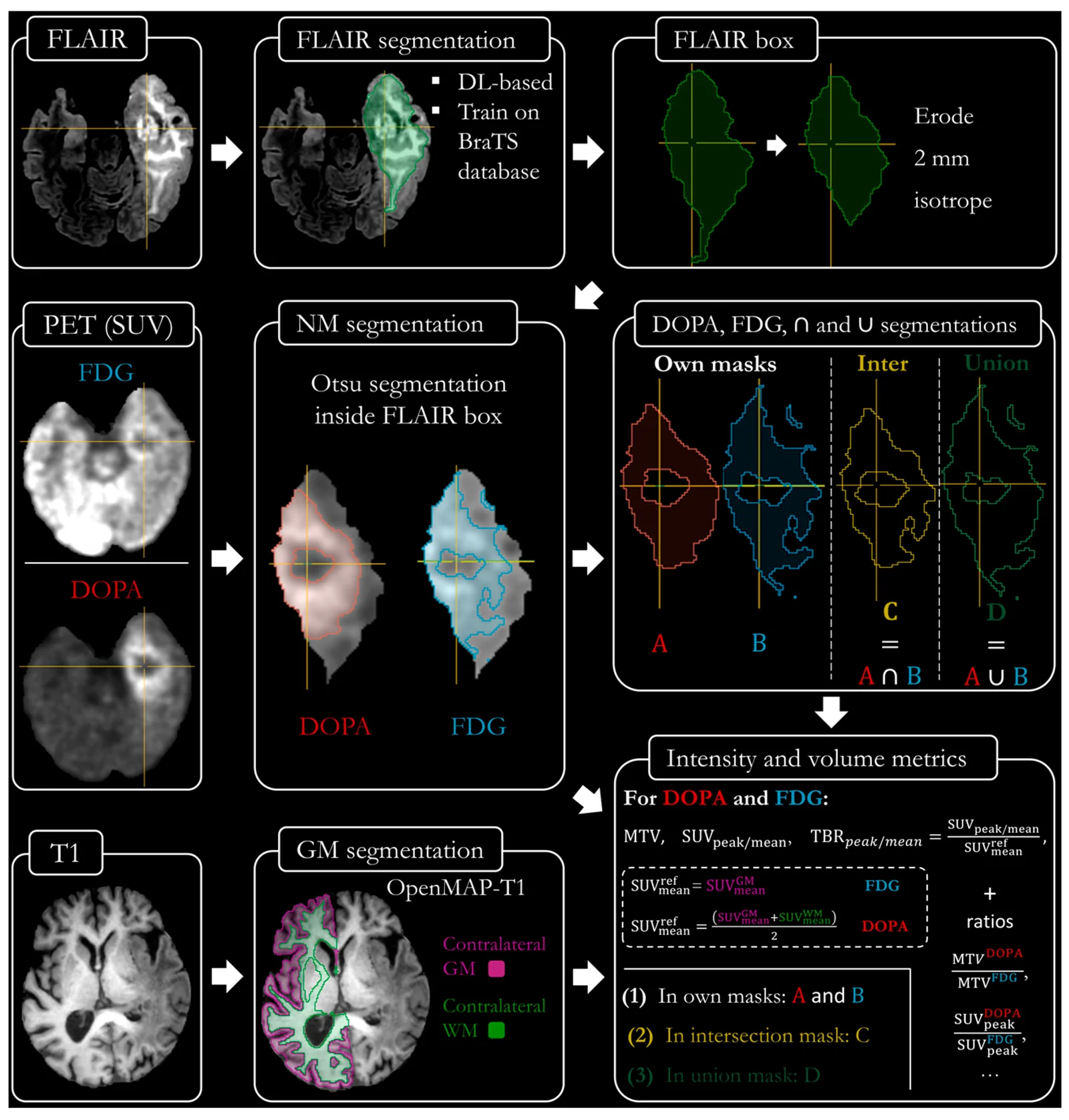
**Multimodal PET/MR image-processing pipeline and PET metric extraction**. Paired [^18^F]FDOPA/[^18^F]FDG PET/MR examinations were rigidly registered into a common spatial frame. FLAIR MRI was segmented with a BraTS-trained deep-learning model, then eroded by 2 mm to define the FLAIR box for PET segmentation. FDOPA and FDG masks were independently generated by Otsu thresholding, yielding tracer-specific masks, their intersection, and their union. OpenMAP-T1 provided contralateral reference regions for TBR calculation. Intensity metrics, MTV, and FDOPA/FDG ratios were extracted across all spatial domains.

Within these domains, PET metrics included intensity-based measures (SUV_mean_, SUV_peak_, TBR_mean_, and TBR_peak_) and the volume-based measure MTV. TBR_mean_ and TBR_peak_ were defined as SUV_mean_/SUV_ref_ and SUV_peak_/SUV_ref_, respectively, where SUV_ref_ was measured in the contralateral gray matter for FDG and in contralateral gray-plus-white matter for FDOPA. For each domain, FDOPA- and FDG-derived values were computed, and FDOPA/FDG ratios were calculated for each corresponding metric. In tracer-specific domains, each tracer was quantified within its own PET tumor mask, whereas in the union and intersection domains both tracers were quantified within the same shared spatial mask. Overall, 42 imaging metrics were derived per examination, including MRI-derived WT volume. The full image-processing pipeline is illustrated in Figure 2.

Throughout the manuscript, the recurrence intensity-balance metric is denoted as the FDOPA/FDG SUV_mean_^union^ ratio, corresponding to the FDOPA-to-FDG SUV_mean_ ratio measured within the shared union mask.

### 2.5. Statistical Analysis

#### 2.5.1. Longitudinal Analyses

Longitudinal analyses were restricted to the paired subgroup with both PET1 and PET2 available. For each metric, within-subject change was defined as Δ = PET2 − PET1. Changes between PET1 and PET2 were assessed separately in the initial resection and biopsy groups using Wilcoxon signed-rank tests, and Δ values were compared between groups using Mann–Whitney U tests. Longitudinal changes were visualized using a signed −log_10_(*p*) heatmap.

#### 2.5.2. Survival Analyses

Overall survival was analyzed separately from PET1 (OS1) and PET2 (OS2). OS1 was measured from the first acquisition date of the paired baseline assessment, whereas OS2 was measured from the later acquisition date of the paired recurrence assessment, ensuring that both recurrence tracer measurements preceded OS2 follow-up. No imputation was performed. Analyses used complete cases for the variables required by each endpoint-specific model; the absence of PET1 or PET2 determined analysis-set membership rather than being imputed.

At each time point, all imaging metrics were first screened using Kaplan–Meier analyses with median dichotomization and univariable Cox proportional hazards models, with hazard ratios expressed per +1 SD. Given the exploratory design, metrics significant in either univariable approach at a given time point were retained for endpoint-specific adjusted modeling. To assess multiplicity, Benjamini–Hochberg false discovery rate (FDR) q-values were calculated separately for the 42 continuous Cox screening tests at OS1 and OS2. These q-values were reported as a sensitivity analysis and did not retrospectively alter the nominal candidate-retention rule; median-split Kaplan–Meier analyses were considered descriptive and were not included in the FDR family.

Each retained metric was then entered individually into adjusted Cox models including age, MGMT methylation status, initial surgery type, and WHO performance status. Continuous covariates were standardized per +1 SD, and volume-based variables were natural-log transformed before standardization. Metrics that remained independently associated with survival after adjustment were selected for downstream analyses. The adjustment set was chosen on clinical prognostic grounds and deliberately limited relative to the 44 OS2 events; no data-driven covariate selection was performed.

Pairwise correlation between selected PET metrics was assessed using Spearman coefficients, and collinearity was evaluated within adjusted models using variance inflation factors (VIF). Model-level discrimination was assessed using Harrell’s concordance index (C-index). Given the exploratory same-cohort model development, C-index differences were treated as descriptive rather than as formal tests of superiority; no hypothesis test of ΔC was performed. Apparent C-indices were interpreted alongside .632 bootstrap-corrected estimates to assess optimism.

Exploratory composite metrics were then constructed from complementary selected metrics by multiplying an intensity or intensity-ratio metric by the natural-log-transformed value of a volume metric. Candidate composites were evaluated using Cox modeling, Kaplan–Meier analysis, C-index, bootstrap .632-corrected C-index, and time-dependent AUC. The final selected composite was illustrated using Kaplan–Meier analysis. IR-MTV was a post hoc exploratory composite restricted to retained OS2 metrics; no fitted weights or automated optimization algorithm were used. Because construction and evaluation occurred in the same cohort, performance estimates remain susceptible to optimism despite bootstrap correction. Statistical analyses were performed using Python 3.11 (Python Software Foundation, Wilmington, DE, USA) with SciPy 1.17.1 and lifelines 0.30.3.

## 3. Results

### 3.1. Patient Characteristics and Analysis Sets

The study cohort included 65 consecutive patients with IDH-wildtype glioblastoma who underwent paired [^18^F]FDOPA and [^18^F]FDG PET at baseline and/or recurrence. Initial management consisted of surgical resection in 42 patients and biopsy in 23 patients (Table 1). Biopsy patients had a higher proportion of WHO performance status ≥ 2 than resection patients, whereas other baseline characteristics were broadly balanced between groups. Thirty-six patients contributed to the baseline survival analysis from PET1 (OS1), 55 to the post-recurrence survival analysis from PET2 (OS2), and 26 had both PET1 and PET2 available for paired longitudinal analysis (Figure 1).

### 3.2. Longitudinal Changes in the Paired Cohort

**Figure 3:**
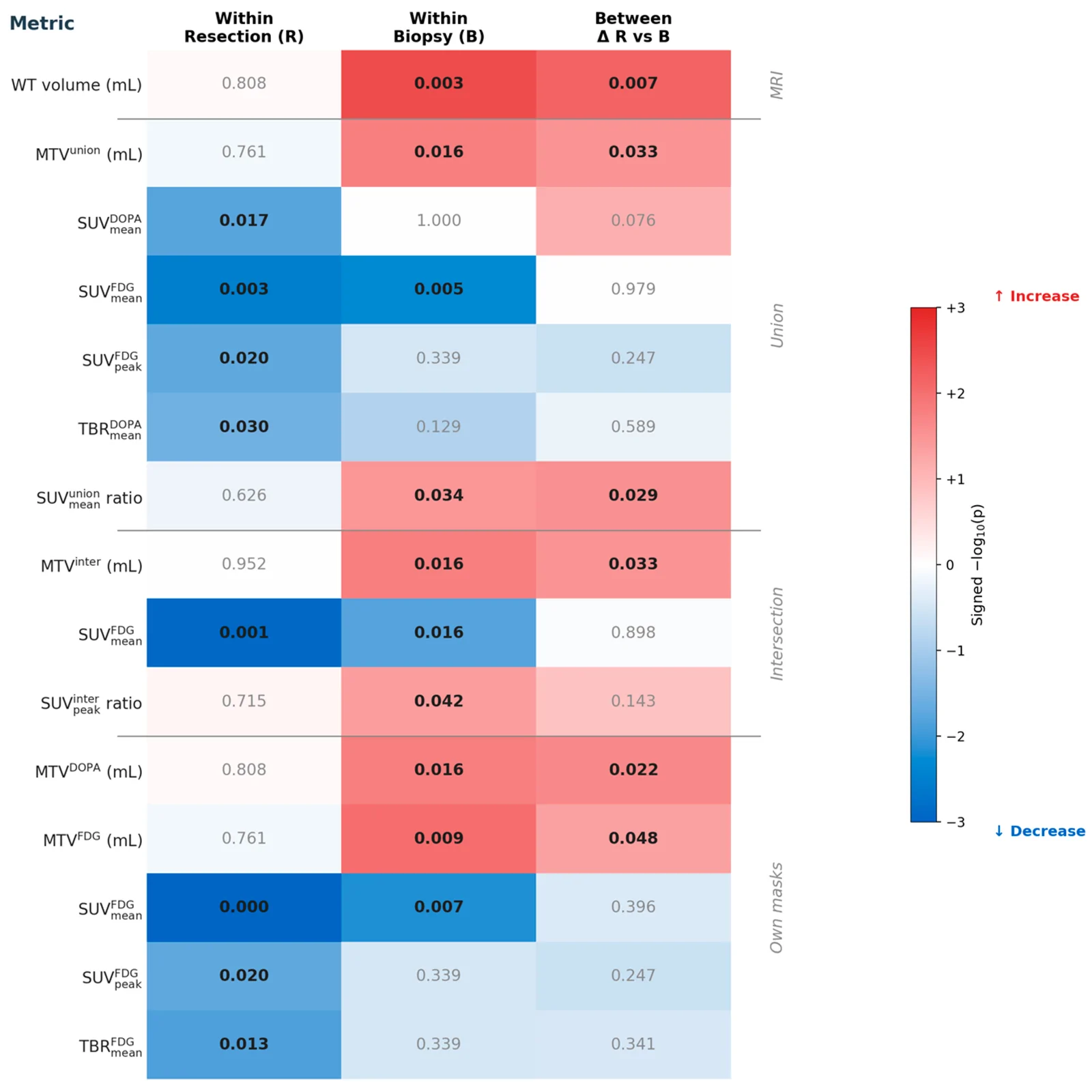
**Longitudinal direction and strength of metric changes in the paired cohort**. Directional heatmap of selected longitudinal PET and MRI metric changes between PET1 and PET2 in the paired cohort (*n* = 26; resection, *n* = 14; biopsy, *n* = 12). Within-group changes were tested using Wilcoxon signed-rank tests, and between-group Δ values using Mann–Whitney U tests. Cell color encodes signed −log_10_(*p*), with red indicating increase, blue decrease, and gray no directional signal. Cell values are *p* values; spatial domains are indicated at right.

Figure 3 summarizes longitudinal paired analyses for metrics showing significant change in the resection and/or biopsy groups; complete results for all tested metrics are reported in Table S1. Volume-related metrics increased after biopsy but not after resection, including WT volume (*p* = 0.003), MTV^union^ (*p* = 0.016), MTV^intersection^ (*p* = 0.016), MTV^DOPA^ (*p* = 0.016), and MTV^FDG^ (*p* = 0.009), with significant between-group differences for these volume metrics (all *p* ≤ 0.048). FDG SUV_mean_ decreased across the union, intersection, and own-mask domains in both groups, whereas additional FDG-derived decreases after resection involved ${\mathrm{SUV}}_{\mathrm{peak}}^{\mathrm{FDG}}$ and ${\mathrm{TBR}}_{\mathrm{mean}}^{\mathrm{FDG}}$. In contrast, FDOPA-derived intensity changes were mainly detected in the union domain, with decreases in ${\mathrm{SUV}}_{\mathrm{mean}}^{\mathrm{DOPA}}$ (*p* = 0.017) and ${\mathrm{TBR}}_{\mathrm{mean}}^{\mathrm{DOPA}}$ (*p* = 0.029) after resection. The FDOPA/FDG ${\mathrm{SUV}}_{\mathrm{mean}}^{\mathrm{union}}$ ratio increased after biopsy (*p* = 0.034) but not after resection (*p* = 0.626), with a significant between-group difference in Δ (*p* = 0.029). Overall, longitudinal changes reflected biopsy-associated volume expansion, broadly decreasing FDG uptake, and union-domain sensitivity to FDOPA intensity and FDOPA-to-FDG balance.

### 3.3. Survival Screening and Adjusted Models

**Figure 4:**
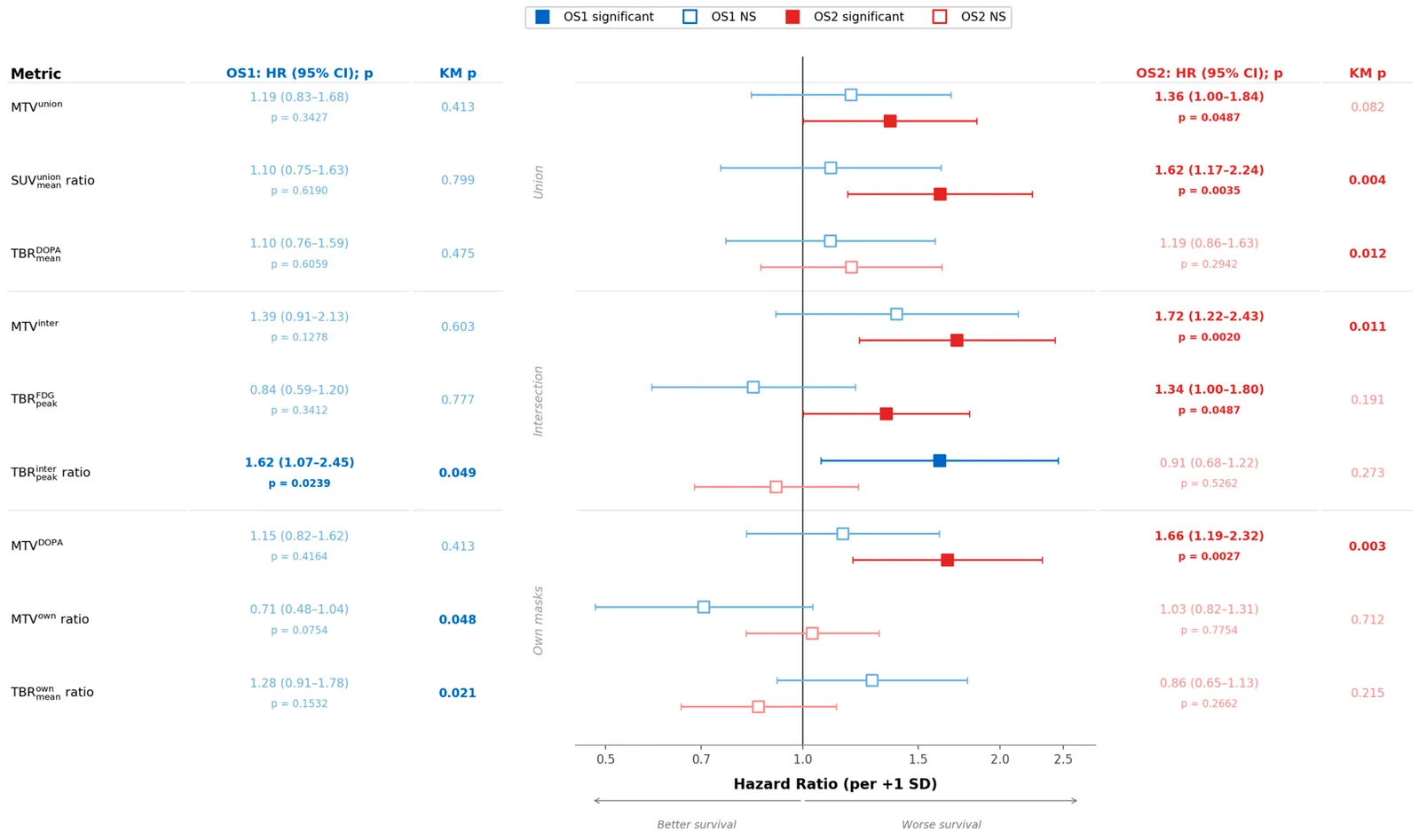
**Selected univariate prognostic screening metrics at baseline and recurrence**. Forest-style summary of PET metrics retained after univariate screening at OS1 or OS2. Hazard ratios are expressed per +1 SD. Filled squares indicate significant Cox associations, open squares non-significant Cox associations, and outer columns show Kaplan–Meier log-rank *p* values. Complete results are reported in Table S2.

Figure 4 summarizes metrics retained after univariate screening, defined as those significant in univariate Cox and/or Kaplan–Meier analyses for OS1 or OS2; complete univariate results are reported in Table S2. At OS1, retained signals were limited to ratio-based metrics: the intersection-domain FDOPA/FDG TBR_peak_ ratio was significant in both univariate Cox and Kaplan–Meier analyses, whereas own-mask FDOPA/FDG MTV and TBR_mean_ ratios were significant by Kaplan–Meier analysis only. At OS2, the prognostic signal was broader. Three recurrence metrics showed a concordant significance in both univariate Cox and Kaplan–Meier analyses: union-based FDOPA/FDG SUV_mean_ ratio (HR, 1.62; *p* = 0.0035; KM *p* = 0.004), MTV^intersection^ (HR, 1.72; *p* = 0.0020; KM *p* = 0.011), and MTV^DOPA^ (HR, 1.66; *p* = 0.0027; KM *p* = 0.003). Benjamini–Hochberg correction of the 42 continuous Cox screening tests yielded q = 0.049 for each of these three OS2 metrics; no OS1 Cox association remained significant after FDR correction.

**Figure 5:**
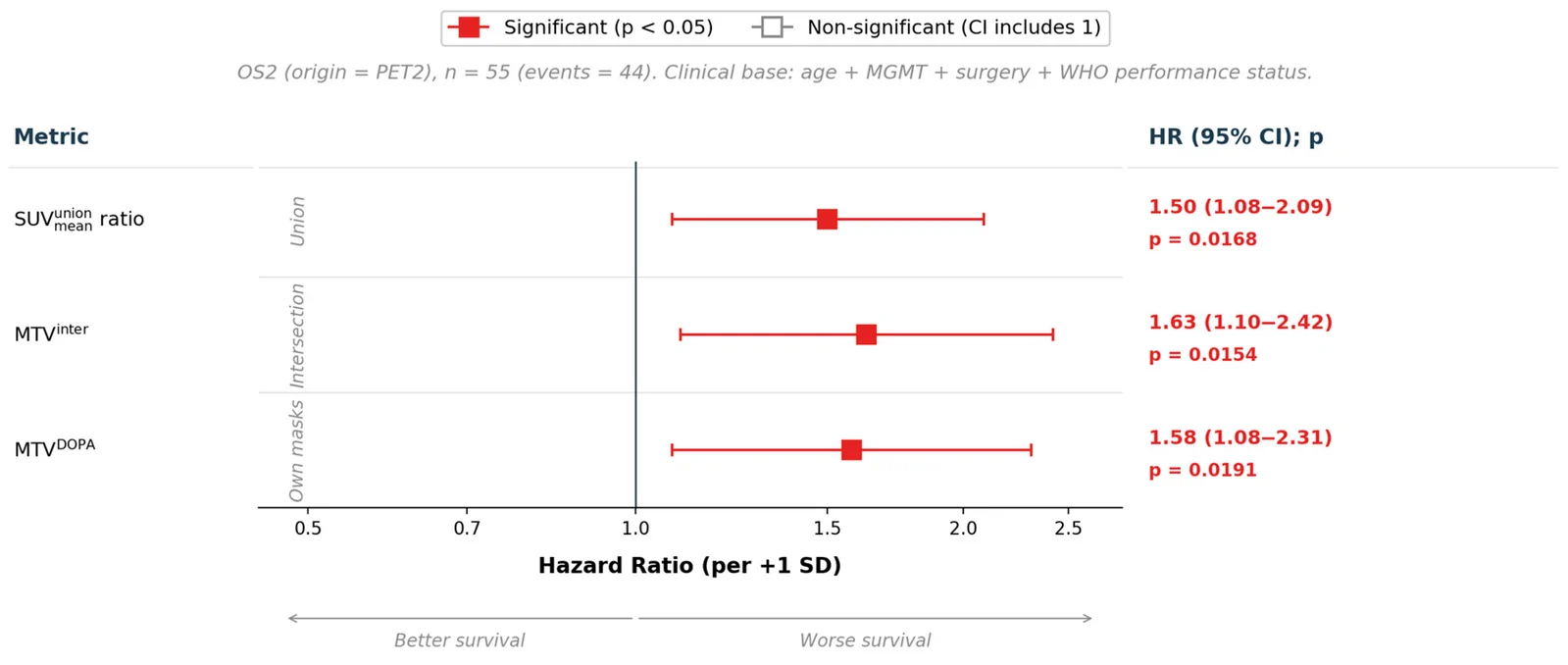
**Adjusted OS2 associations of the 3 retained simple PET metrics**.

Figure 5 shows that these same three OS2 metrics remained independently associated with survival after adjustment for age, MGMT methylation status, initial surgery, and WHO performance status: FDOPA/FDG ${\mathrm{SUV}}_{\mathrm{mean}}^{\mathrm{union}}$ ratio (HR, 1.50; 95% CI, 1.08–2.09; *p* = 0.0168), MTV^intersection^ (HR, 1.63; 95% CI, 1.10–2.42; *p* = 0.0154), and MTV^DOPA^ (HR, 1.58; 95% CI, 1.08–2.31; *p* = 0.0191). Complete adjusted Cox results are reported in Table S3.

In the FDOPA segmentation sensitivity analysis, survival stratification remained directionally concordant when metabolic volume was delineated using the fixed 1.6 × contralateral background threshold (log-rank *p* = 0.025). Five of 55 patients (9.1%) with confirmed recurrence had no measurable FDOPA volume above this fixed threshold.

Forest plot of the three recurrence PET metrics that remained significantly associated with OS2 after adjustment for age, MGMT methylation status, initial surgery, and WHO performance status (*n* = 55, 44 events). Hazard ratios are expressed per +1 SD. Retained metrics captured two dimensions: inter-tracer balance, represented by the FDOPA/FDG ${\mathrm{SUV}}_{\mathrm{mean}}^{\mathrm{union}}$ ratio, and metabolic burden, represented by MTV^intersection^ and MTV^DOPA^. Complete adjusted results are reported in Table S3.

### 3.4. Complementarity and Intensity-Ratio-MTV (IR-MTV) Composite Performance

Spearman analysis showed that the FDOPA/FDG ${\mathrm{SUV}}_{\mathrm{mean}}^{\mathrm{union}}$ ratio had low correlation with MTV^DOPA^ (ρ = 0.33) and MTV^intersection^ (ρ = 0.38), whereas MTV^DOPA^ and MTV^intersection^ were highly correlated (ρ = 0.97), indicating redundancy between volume metrics but complementarity between intensity balance and tumor burden. To explore whether this complementarity was associated with higher model discrimination, retained PET metrics were then added to the clinical base model individually and in combination. Adding the FDOPA/FDG ${\mathrm{SUV}}_{\mathrm{mean}}^{\mathrm{union}}$ ratio alone yielded a higher apparent C-index, from 0.668 to 0.701, and adding MTV^DOPA^ alone yielded an apparent C-index of 0.685. The model combining the FDOPA/FDG ${\mathrm{SUV}}_{\mathrm{mean}}^{\mathrm{union}}$ ratio and MTV^DOPA^ yielded the highest apparent discrimination (C-index, 0.738; ΔC = +0.070; max VIF = 1.39), supporting exploration of a single composite metric. Complete model-level results are reported in Table S4. Because this two-component model showed the highest apparent discrimination, the corresponding FDOPA-specific composite was retained as the primary IR-MTV metric, whereas the intersection-volume composite was evaluated as a sensitivity analysis.

The corresponding composite, IR-MTV^DOPA^, was defined as FDOPA/FDG ${\mathrm{SUV}}_{\mathrm{mean}}^{\mathrm{union}}$ ratio × log(MTV^DOPA^).

Table 2 summarizes the performance of IR-MTV^DOPA^ and its component metrics. IR-MTV^DOPA^ showed a stronger OS2 association and higher apparent discrimination than either component alone, with HR 1.73 (95% CI, 1.23–2.45; *p* = 0.0018), log-rank *p* = 0.0001, C-index 0.730, bootstrap .632-corrected C-index 0.684, and 12-month AUC 0.863.

**Figure 6:**
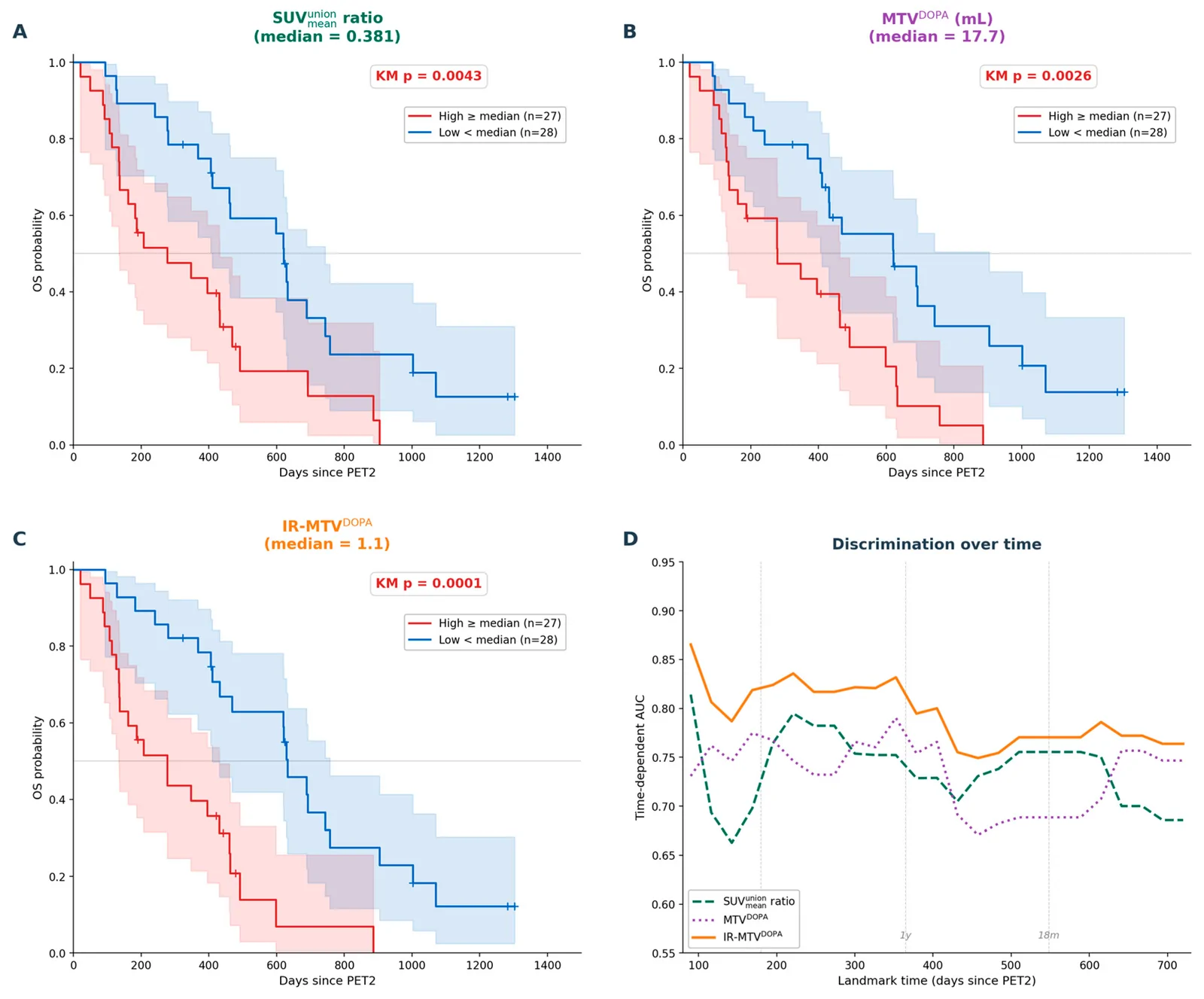
**Exploratory performance of IR-MTV^DOPA^ versus its component metrics for OS2**. Kaplan–Meier curves for OS2 according to median-dichotomized FDOPA/FDG ${\mathrm{SUV}}_{\mathrm{mean}}^{\mathrm{union}}$ (**A**), MTV^DOPA^ (**B**), and IR-MTV^DOPA^ (**C**). IR-MTV^DOPA^ was defined as FDOPA/FDG ${\mathrm{SUV}}_{\mathrm{mean}}^{\mathrm{union}}$ ratio × log(MTV^DOPA^). (**D**) shows time-dependent AUC profiles over follow-up. OS2 originated from recurrence PET2 (*n* = 55, 44 events). Reference and composite metrics are summarized in Table 2.

Figure 6 illustrates the corresponding Kaplan–Meier curves and time-dependent AUC profiles, showing stronger apparent survival separation and higher time-dependent AUC values for IR-MTV^DOPA^ than for the FDOPA/FDG ${\mathrm{SUV}}_{\mathrm{mean}}^{\mathrm{union}}$ ratio or MTV^DOPA^ alone.

## 4. Discussion

In this study, we analyzed paired, spatially aligned [^18^F]FDOPA/[^18^F]FDG PET/MR examinations to quantify intensity, volume, and inter-tracer ratio metrics across tracer-specific, intersection, and union PET domains, and to assess their longitudinal evolution and survival associations at baseline and recurrence in IDH-wildtype glioblastoma. The main findings were 3-fold. First, prognostic associations were sparse at baseline and more prominent at recurrence. Second, recurrence-associated PET metrics captured two complementary dimensions: FDOPA-to-FDG intensity balance and metabolic tumor burden. Third, integrating these dimensions into the exploratory IR-MTV^DOPA^ metric showed higher apparent post-recurrence survival discrimination than either dimension alone. These findings suggest that a spatially aligned paired [^18^F]FDOPA/[^18^F]FDG PET/MR framework can integrate inter-tracer intensity balance and metabolic tumor burden as a candidate framework for post-recurrence risk stratification in IDH-wildtype glioblastoma.

The differing OS1 and OS2 screening patterns are consistent with, but do not establish, metabolic remodeling at recurrence. Recurrent glioblastoma is shaped by treatment, clonal selection, and adaptation [25,26]. The paired longitudinal analysis in the 26 patients with both time points showed biopsy-associated expansion of volume-related metrics, broadly decreasing FDG uptake, and a union-domain shift in the FDOPA-to-FDG balance. These within-patient observations are compatible with remodeling of the PET-defined tumor phenotype but should not be used to infer a direct survival comparison between the OS1 and OS2 analysis sets.

The recurrence survival signal was not explained by a single PET readout. After adjustment, three metrics remained associated with OS2: the FDOPA/FDG ${\mathrm{SUV}}_{\mathrm{mean}}^{\mathrm{union}}$ ratio (HR, 1.50; *p* = 0.0168), MTV^DOPA^ (HR, 1.58; *p* = 0.0191), and MTV^intersection^ (HR, 1.63; *p* = 0.0154). The union-based SUV_mean_ ratio showed limited correlation with the volume metrics, whereas MTV^DOPA^ and MTV^intersection^ were highly correlated. This supports a 2-dimensional structure in which the FDOPA-to-FDG intensity balance and metabolic tumor burden provide partially complementary prognostic information.

Although the intersection domain may better capture tissue jointly identified by both tracers, the union domain may be more informative for inter-tracer ratios because it retains tracer-discordant regions. This broader spatial support may better reflect FDOPA-to-FDG imbalance across the PET-defined tumor habitat. In contrast, MTV^DOPA^ and MTV^intersection^ appear to capture a shared burden-related dimension.

The exploratory IR-MTV^DOPA^ metric was designed to integrate these two dimensions by weighting FDOPA-to-FDG intensity balance by metabolic tumor burden. This integration showed higher apparent model discrimination than the clinical base model and the individual PET metrics. In model-level analyses, combining the union-based SUV_mean_ ratio with MTV^DOPA^ yielded a higher apparent C-index than either metric alone. As a single composite readout, IR-MTV^DOPA^ showed stronger apparent OS2 stratification than both the SUV_mean_ ratio and MTV^DOPA^ alone, with a log-rank *p* value of 0.0001, an adjusted Cox HR of 1.73, an apparent C-index of 0.730, a bootstrap .632-corrected C-index of 0.684, and a 12-month AUC of 0.863.

This complementarity is biologically plausible. Due to their genetic instability and high proliferation rate, cancer cells develop a wide range of metabolic phenotypes and evolve in unpredictable ways. This metabolic diversity is driven by environmental pressures, primarily vascularization and oxygen supply, as well as other factors such as treatment. While a large proportion of the tumor responds to treatment, subclones with distinct metabolic phenotypes may resist therapy, expand, and ultimately drive treatment-resistant recurrence [27,28,29]. One hypothesis raised by these imaging findings is that, despite an overall longitudinal decrease in FDG SUVmean, poor-prognosis relapse may combine a larger FDOPA-positive metabolic burden with persistent FDG-defined activity. This pattern is compatible with treatment-selected metabolic heterogeneity at recurrence [26,30]. However, the present PET data do not establish a specific cellular or molecular phenotype, and this biological interpretation remains hypothesis-generating without spatially matched tissue validation.

Previous FDOPA studies in recurrent glioma have reported diagnostic and, in some series, prognostic associations, including for MTV-based metrics, but the evidence remains heterogeneous and largely based on single-tracer analyses [9,10,11]. Consistently, our recent systematic review found that prognostic PET evidence in high-grade glioma remains uneven across tracers, metrics, populations, and endpoints [12]. In this context, the added value of the present study is not to reintroduce MTV^DOPA^ as an isolated marker, but to position it within a paired-tracer framework showing that tumor burden and FDOPA-to-FDG intensity balance provide complementary recurrence-risk information. Preoperative FDOPA uptake has also shown prognostic associations in treatment-naive glioma cohorts [31].

From a translational perspective, MTV^DOPA^ remains the most directly implementable metric because it relies on a single amino-acid tracer and a volumetric readout aligned with emerging PET response frameworks [15,16]. However, the higher apparent discrimination of IR-MTV^DOPA^ suggests that paired-tracer PET/MR may provide recurrence-risk information beyond the amino-acid tumor burden alone. If externally validated, this framework could help identify patients with higher-risk recurrent disease who may require more aggressive or alternative therapeutic strategies. Clinical implementation would require access to both tracers, two radiotracer administrations and acquisitions, robust inter-scan registration, and standardized segmentation; these requirements may limit feasibility outside centers with established dual-tracer workflows.

The segmentation sensitivity analysis showed concordant OS2 stratification when a fixed 1.6 × FDOPA background threshold was used, although 9.1% of confirmed recurrences yielded no measurable volume with this approach. This finding illustrates a practical limitation of fixed-threshold volumetry in this cohort and supports the use of the symmetric Otsu-based framework for the primary paired-tracer analyses, while confirming that the observed prognostic direction was not specific to a single FDOPA delineation strategy.

This study has limitations. It was retrospective, single-center, and modest in size, particularly for OS1 and subgroup longitudinal analyses. Paired PET/MR examinations were acquired as separate scans within a short interval rather than during a single session. The statistical workflow involved exploratory screening of multiple correlated imaging metrics and exploratory composite construction, and no external validation cohort was available; the resulting associations should therefore be considered hypothesis-generating. Finally, no spatially matched histologic or molecular validation was available to assign biological states to tracer-defined compartments. External validation is required before clinical implementation. As with all PET-derived volumetric biomarkers, delineated volumes remain dependent on segmentation strategy, background definition, image resolution, and lesion-to-background contrast [32].

Additional limitations affect interpretation. PET contributed to routine recurrence assessment, creating potential incorporation bias because recurrence confirmation was not independent of the imaging under study. OS1 and OS2 were partially overlapping analysis sets, with only 26 patients contributing both time points; differences in screening patterns between baseline and recurrence therefore cannot be interpreted as a formal within-patient survival comparison. With 55 patients and 44 events at OS2, adjusted estimates and especially the exploratory composite remain susceptible to statistical uncertainty, model instability, and optimism. Residual confounding is also possible, including from recurrence-directed treatments not included in the adjustment set. Finally, the large number of correlated imaging metrics increases the risk of type I error inflation; FDR-adjusted q-values address this for the continuous Cox screening stage, whereas the longitudinal and median-split Kaplan–Meier analyses remain exploratory.

## 5. Conclusions

In IDH-wildtype glioblastoma, paired [^18^F]FDOPA/[^18^F]FDG PET showed prognostic associations that were more prominent at recurrence than in baseline screening. At recurrence, inter-tracer intensity balance and metabolic tumor burden provided partially complementary survival information. Their exploratory integration into IR-MTV^DOPA^ showed higher apparent post-recurrence discrimination in this cohort, supporting further evaluation of paired-tracer PET metrics for recurrence risk stratification. External validation is required before clinical implementation.

## Figures and Tables

**Table 1:** **Baseline characteristics and timing relative to progression, by initial surgical approach**. Demographic, clinical, molecular, timing, and outcome characteristics for the full cohort and by initial surgery type. Continuous variables are shown as median [IQR] and categorical variables as *n* (%). *p* values compare resection and biopsy groups.

Characteristic	Total (*n* = 65)	Resection (*n* = 42)	Biopsy (*n* = 23)	*p*
**Demographics/Clinical**				
Patients, *n*	65	42	23	
Male sex, *n* (%)	38 (58.5%)	27 (64.3%)	11 (47.8%)	0.293
Age at initial surgery, years, median [IQR]	63.5 [56.2–69.2]	61.8 [53.7–66.7]	65.2 [59.5–72.2]	0.063
Left-sided component, *n* (%)	33 (50.8%)	19 (45.2%)	14 (60.9%)	0.301
WHO performance status ≥ 2, *n* (%)	20 (30.8%)	9 (21.4%)	11 (47.8%)	**0.048**
**Molecular**				
MGMT methylated, *n* (%)	31 (47.7%)	17 (40.5%)	14 (60.9%)	0.129
**Timing/Clinical Context**				
Interval baseline PET → recurrence PET, days, median [IQR]	273.5 [210.5–399.8] (*n* = 26)	326.5 [233.2–399.8] (*n* = 14)	263.0 [170.2–338.0] (*n* = 12)	0.303
**Outcomes**				
Overall survival from baseline PET (OS1), days, median [IQR]	517.0 [268.5–857.0] (*n* = 36)	697.0 [508.0–953.0] (*n* = 17)	360.0 [129.0–602.5] (*n* = 19)	0.005
Overall survival from recurrence PET (OS2), days, median [IQR]	408.0 [181.0–626.0] (*n* = 55)	440.0 [298.0–660.0] (*n* = 39)	186.5 [109.2–448.0] (*n* = 16)	0.016
Deaths (events), *n* (%)	51 (78.5%)	30 (71.4%)	21 (91.3%)	0.112

Values are shown as median [IQR] for continuous variables and *n* (%) for categorical variables. For imaging and timing metrics, *n* available is reported in parentheses when <total. *p*-values: Fisher’s exact test (categorical) and Mann–Whitney U test (continuous). WHO performance status was used as ordinal (0–3) in multivariable models (Tables S3 and S4).

**Table 2:** **Reference recurrence-associated metrics and exploratory IR-MTV^DOPA^/IR-MTV^inter^ composites for OS2**. Kaplan–Meier, adjusted Cox, and discrimination performance for OS2 recurrence metrics and exploratory IR-MTV composites. Cox models were adjusted for age, MGMT methylation status, initial surgery, and WHO performance status. HRs are per +1 SD; volumes were log-transformed before standardization.

Metric	KM log-Rank *p*	Cox HR (95% CI)	Cox *p*	C-Index	C.632	AUC 12 m
Reference metrics retained after multivariable adjustment						
SUV_mean_^union^ ratio	0.0043	1.50 (1.08–2.09)	0.0168	0.701	0.653	0.816
MTV^DOPA^ (mL)	0.0026	1.58 (1.08–2.31)	0.0191	0.685	0.642	0.816
MTV^inter^ (mL)	0.0110	1.63 (1.10–2.42)	0.0154	0.689	0.649	0.820
IR-MTV^DOPA^ (composite: intensity ratio × log volume)						
IR-MTV^DOPA^: SUV_mean_^union^ ratio × log(MTV^DOPA^)	0.0001	1.73 (1.23–2.45)	0.0018	0.730	0.684	0.863
IR-MTV^inter^ (sensitivity composite: alternative volume term)						
IR-MTV^inter^: SUV_mean_^union^ ratio × log(MTV^inter^)	0.0009	1.74 (1.23–2.45)	0.0017	0.732	0.685	0.867

## Data Availability

The anonymized patient-level clinical and quantitative imaging data supporting the findings of this study are available from the corresponding author upon reasonable request. Raw imaging data are not publicly available because of patient privacy and institutional restrictions.

## Supplementary Materials

The following supporting information can be downloaded at: https://www.mdpi.com/article/10.3390/cancers18203333/s1, Table S1, longitudinal changes in MRI tumor volume and PET metrics by spatial domain; Table S2, univariate Kaplan–Meier and Cox analyses for all PET imaging metrics at baseline and recurrence; Table S3, multivariable Cox proportional hazards models for OS1 and OS2; Table S4, stepwise derivation of the IR-MTV composite. Figure S1: Chemical structures of [^18^F]FDG and [^18^F]FDOPA.

## Abbreviations

The following abbreviations are used in this manuscript:

AUC	area under the curve
CI	confidence interval
FDG	fluorodeoxyglucose
FDOPA	fluorodihydroxyphenylalanine
FLAIR	fluid-attenuated inversion recovery
HR	hazard ratio
IDH	isocitrate dehydrogenase
IR-MTV	intensity-ratio–metabolic tumor volume
KM	Kaplan–Meier
MGMT	O6-methylguanine-DNA methyltransferase
MRI	magnetic resonance imaging
MTV	metabolic tumor volume
OS	overall survival
PET	positron emission tomography
TBR	tumor-to-background ratio
VIF	variance inflation factor
WT	whole tumor
WHO	World Health Organization
