# Supplementary material for: Paired [18F]FDOPA and [18F]FDG PET Shows Complementary Prognostic Associations at Recurrence in IDH-Wildtype Glioblastoma
Source: Cancers (Basel). 2026 Oct 15;18(20):3333. doi: 10.3390/cancers18203333 (PMC13702550; doi:10.3390/cancers18203333)
Supplement: Supplementary file 1 [file cancers-18-03333-s001.zip › Figure S1 .pdf]

A **B**

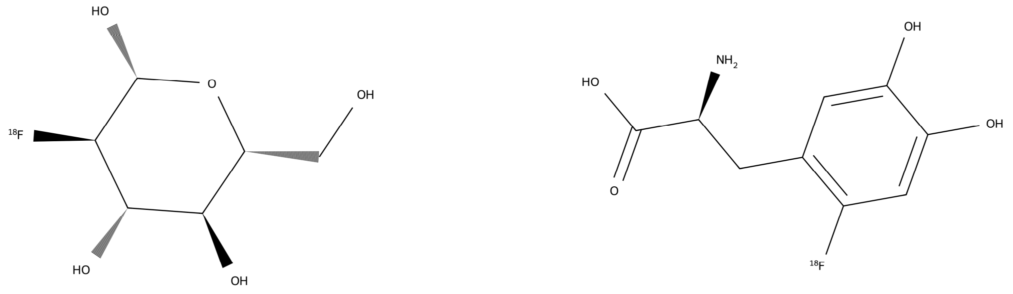

2-deoxy-2-[^{18}F]fluoro-D-glucose
([^{18}F]FDG)

6-[^{18}F]fluoro-L-3,4-dihydroxyphenylalanine
(6-[^{18}F]FDOPA)

Figure S1: Chemical structures of [^{18}F]FDG and [^{18}F]FDOPA
