# Supplementary material for: Paired [18F]FDOPA and [18F]FDG PET Shows Complementary Prognostic Associations at Recurrence in IDH-Wildtype Glioblastoma
Source: Cancers (Basel). 2026 Oct 15;18(20):3333. doi: 10.3390/cancers18203333 (PMC13702550; doi:10.3390/cancers18203333)
Supplement: Supplementary file 1 [file cancers-18-03333-s001.zip › Table_S1.pdf]

Table S1. Longitudinal changes in MRI tumor volume and PET metrics by spatial domain (paired cohort, n = 26; 2-mm erosion).

Values are median [IQR]. Δ = (PET2 − PET1). Within-group: paired Wilcoxon signed-rank test. Between-group: Mann–Whitney U on Δ. Volumes in mL. TBRmean = MeanSUV/reference; TBRpeak = PeakSUV/reference (CM for FDG, CM_WM for DOPA). Ratio = DOPA/FDG. Bold red: p < 0.05.

Metric	n (R)	n (B)	Resection (R)				Biopsy (B)				p(Δ R vs B)
			PET1	PET2	Δ	p	PET1	PET2	Δ	p	
**MRI-based**											
WT volume (mL)	14	12	62.3 [33.2–155.2]	73.3 [35.7–115.9]	+11.5 [−40.5–28.6]	0.8077	47.6 [38.2–95.0]	141.4 [87.9–162.4]	+61.5 [26.7–104.5]	**0.0034**	**0.0069**
**Union (A∪B)**											
MTV^{union} (mL)	14	12	19.1 [10.0–38.1]	21.1 [9.7–39.6]	−2.6 [−11.9–10.5]	0.7609	17.7 [10.9–37.8]	42.1 [31.2–50.5]	+24.9 [4.3–28.9]	**0.0161**	**0.0328**
SUV_{mean}^{DOPA}	14	12	1.87 [1.54–2.26]	1.41 [1.20–1.51]	−0.36 [−0.76–−0.14]	**0.0166**	1.80 [1.19–2.15]	1.54 [1.35–1.82]	+0.07 [−0.31–0.34]	1.0000	0.0760
SUV_{mean}^{FDG}	14	12	4.78 [3.68–5.96]	4.03 [3.28–4.39]	−0.56 [−2.17–−0.30]	**0.0031**	5.02 [4.19–5.42]	3.93 [3.55–4.66]	−0.89 [−1.46–−0.37]	**0.0049**	0.9795
SUV_{mean}^{union} ratio	14	12	0.39 [0.31–0.48]	0.37 [0.29–0.49]	−0.01 [−0.08–0.04]	0.6257	0.36 [0.24–0.41]	0.44 [0.30–0.51]	+0.10 [0.04–0.15]	**0.0342**	**0.0288**
SUV_{peak}^{DOPA}	14	12	2.56 [2.31–3.36]	2.08 [1.93–3.12]	−0.32 [−0.76–0.06]	0.1726	2.75 [1.77–3.13]	2.52 [2.27–3.00]	+0.07 [−0.44–1.20]	0.3804	0.1292
SUV_{peak}^{FDG}	14	12	7.01 [6.12–9.05]	5.94 [5.04–7.01]	−0.90 [−1.81–−0.37]	**0.0203**	6.53 [6.01–7.47]	5.88 [5.44–7.52]	−0.34 [−0.96–0.30]	0.3394	0.2472
SUV_{peak}^{union} ratio	14	12	0.40 [0.28–0.52]	0.36 [0.32–0.50]	+0.03 [−0.06–0.09]	0.6698	0.38 [0.33–0.43]	0.44 [0.36–0.64]	+0.10 [0.02–0.15]	0.0522	0.1292
TBR_{mean}^{DOPA}	14	12	2.31 [1.80–2.49]	1.85 [1.48–2.08]	−0.44 [−0.81–−0.07]	**0.0295**	1.99 [1.47–2.60]	1.78 [1.47–1.91]	−0.16 [−0.83–0.05]	0.1294	0.5892
TBR_{mean}^{FDG}	14	12	0.67 [0.57–0.85]	0.58 [0.52–0.69]	−0.10 [−0.21–0.01]	0.0906	0.72 [0.66–0.77]	0.66 [0.57–0.75]	−0.07 [−0.19–−0.00]	0.2661	0.6620
TBR_{mean}^{union} ratio	14	12	3.23 [2.80–3.63]	2.98 [2.49–3.44]	−0.32 [−0.56–0.40]	0.6698	2.63 [2.00–3.11]	2.40 [2.19–3.16]	+0.18 [−0.76–0.39]	0.9697	0.9385
TBR_{peak}^{DOPA}	14	12	3.19 [2.67–3.77]	2.88 [2.57–3.03]	−0.33 [−0.93–0.20]	0.2166	3.11 [2.17–3.57]	2.75 [2.37–3.96]	+0.09 [−0.67–0.40]	0.9697	0.3961
TBR_{peak}^{FDG}	14	12	1.00 [0.87–1.20]	0.88 [0.84–0.98]	−0.06 [−0.24–0.01]	0.1040	0.98 [0.90–1.09]	1.03 [0.93–1.14]	−0.02 [−0.09–0.17]	0.6221	0.1427
TBR_{peak}^{union} ratio	14	12	3.06 [2.87–3.41]	3.17 [2.82–3.58]	−0.08 [−0.42–0.72]	0.7148	2.91 [2.36–4.06]	2.62 [2.41–3.47]	+0.09 [−0.58–0.21]	0.6772	0.4875
**Intersection (A∩B)**											
MTV^{inter} (mL)	14	12	11.0 [3.7–21.6]	12.0 [6.5–18.9]	+0.1 [−5.7–3.5]	0.9515	11.6 [4.0–18.9]	24.7 [15.8–31.5]	+12.8 [4.0–19.9]	**0.0161**	**0.0328**
SUV_{mean}^{DOPA}	14	12	2.23 [1.68–3.05]	1.76 [1.57–1.96]	−0.21 [−0.98–0.05]	0.0785	2.13 [1.43–2.86]	1.77 [1.63–2.13]	−0.17 [−0.39–0.24]	0.7910	0.3681
SUV_{mean}^{FDG}	14	12	5.70 [4.57–6.63]	4.39 [3.72–4.97]	−0.72 [−2.04–−0.19]	**0.0012**	5.39 [4.51–5.93]	4.32 [3.85–4.99]	−0.76 [−1.57–−0.26]	**0.0161**	0.8977
SUV_{mean}^{inter} ratio	14	12	0.41 [0.30–0.50]	0.42 [0.37–0.52]	+0.02 [−0.05–0.09]	0.7148	0.37 [0.34–0.44]	0.46 [0.37–0.52]	+0.10 [−0.01–0.15]	0.0640	0.2917
SUV_{peak}^{DOPA}	14	12	2.55 [2.15–3.36]	2.08 [1.93–3.12]	−0.17 [−0.76–0.48]	0.3575	2.75 [1.77–3.13]	2.52 [2.27–3.00]	+0.07 [−0.44–1.20]	0.3804	0.1729
SUV_{peak}^{FDG}	14	12	6.48 [5.46–7.28]	5.63 [4.86–6.56]	−0.59 [−1.61–0.36]	0.1937	6.53 [5.77–7.47]	5.84 [5.29–7.52]	−0.13 [−0.96–0.30]	0.4697	0.8170
SUV_{peak}^{inter} ratio	14	12	0.40 [0.28–0.52]	0.41 [0.34–0.50]	+0.03 [−0.07–0.10]	0.7148	0.38 [0.33–0.43]	0.45 [0.39–0.64]	+0.11 [0.04–0.17]	**0.0425**	0.1427
TBR_{mean}^{DOPA}	14	12	2.58 [2.05–2.99]	2.43 [1.74–2.69]	−0.39 [−0.90–0.21]	0.1726	2.55 [1.70–3.17]	2.13 [1.67–2.48]	−0.52 [−1.07–0.17]	0.0771	0.7773
TBR_{mean}^{FDG}	14	12	0.76 [0.68–0.91]	0.66 [0.55–0.79]	−0.12 [−0.25–0.02]	0.0580	0.77 [0.69–0.91]	0.72 [0.63–0.80]	−0.10 [−0.19–−0.00]	0.3394	0.6251
TBR_{mean}^{inter} ratio	14	12	3.19 [2.80–3.78]	3.32 [3.07–3.83]	+0.20 [−0.78–1.20]	0.5830	2.82 [2.48–4.21]	2.58 [2.37–3.38]	−0.14 [−0.77–0.35]	0.4238	0.3681
TBR_{peak}^{DOPA}	14	12	3.06 [2.57–3.51]	2.88 [2.57–3.03]	−0.17 [−0.73–0.40]	0.4631	3.11 [2.17–3.57]	2.75 [2.37–3.96]	+0.09 [−0.67–0.40]	0.9697	0.6620
TBR_{peak}^{FDG}	14	12	0.89 [0.80–1.17]	0.85 [0.78–0.94]	−0.04 [−0.21–0.08]	0.4263	0.98 [0.86–1.09]	1.00 [0.91–1.14]	−0.01 [−0.15–0.18]	0.7910	0.3681
TBR_{peak}^{inter} ratio	14	12	3.09 [2.87–3.65]	3.28 [2.82–3.96]	+0.28 [−0.77–0.98]	0.6698	2.91 [2.47–4.16]	2.63 [2.49–3.47]	+0.03 [−0.58–0.51]	0.9097	0.5542
**Own masks**											
MTV^{DOPA} (mL)	14	12	14.2 [6.3–32.7]	16.9 [8.3–26.8]	+0.5 [−7.9–4.8]	0.8077	13.9 [4.6–26.0]	32.8 [21.9–39.7]	+20.1 [6.2–24.4]	**0.0161**	**0.0221**
MTV^{FDG} (mL)	14	12	15.0 [8.3–26.3]	15.7 [8.1–34.1]	−0.1 [−6.3–8.3]	0.7609	14.8 [10.8–31.3]	31.9 [25.3–46.3]	+15.6 [2.1–23.3]	**0.0093**	**0.0477**
MTV^{own} ratio	14	12	1.0 [0.7–1.4]	0.9 [0.7–1.0]	−0.1 [−0.8–0.1]	0.2412	0.9 [0.7–1.1]	0.9 [0.8–1.0]	+0.0 [−0.1–0.3]	0.4238	0.1729
SUV_{mean}^{DOPA}	14	12	2.10 [1.63–2.95]	1.65 [1.47–1.94]	−0.22 [−0.91–0.03]	0.1040	2.04 [1.40–2.74]	1.71 [1.57–2.04]	−0.10 [−0.44–0.26]	0.6772	0.4253
SUV_{mean}^{FDG}	14	12	5.62 [4.49–6.47]	4.09 [3.63–4.64]	−1.03 [−2.24–−0.46]	**0.00037**	5.22 [4.31–5.87]	4.12 [3.68–4.92]	−0.74 [−1.58–−0.34]	**0.0068**	0.3961
SUV_{mean}^{own} ratio	14	12	0.38 [0.27–0.54]	0.42 [0.37–0.54]	+0.03 [−0.04–0.14]	0.3258	0.37 [0.34–0.46]	0.48 [0.37–0.53]	+0.09 [−0.05–0.17]	0.1099	0.4875
SUV_{peak}^{DOPA}	14	12	2.56 [2.31–3.36]	2.08 [1.93–3.12]	−0.32 [−0.76–0.06]	0.1726	2.75 [1.77–3.13]	2.52 [2.27–3.00]	+0.07 [−0.44–1.20]	0.3804	0.1292
SUV_{peak}^{FDG}	14	12	7.01 [6.12–9.05]	5.94 [5.04–7.01]	−0.90 [−1.81–−0.37]	**0.0203**	6.53 [6.01–7.47]	5.88 [5.44–7.52]	−0.34 [−0.96–0.30]	0.3394	0.2472
SUV_{peak}^{own} ratio	14	12	0.40 [0.28–0.52]	0.36 [0.32–0.50]	+0.03 [−0.06–0.09]	0.6698	0.38 [0.33–0.43]	0.44 [0.36–0.64]	+0.10 [0.02–0.15]	0.0522	0.1292
TBR_{mean}^{DOPA}	14	12	2.48 [1.87–2.83]	2.26 [1.69–2.59]	−0.32 [−0.79–0.18]	0.1726	2.49 [1.62–3.11]	2.02 [1.65–2.38]	−0.52 [−1.06–0.17]	0.0771	0.5203
TBR_{mean}^{FDG}	14	12	0.75 [0.67–0.96]	0.59 [0.54–0.75]	−0.15 [−0.28–−0.00]	**0.0134**	0.74 [0.68–0.84]	0.70 [0.62–0.78]	−0.08 [−0.20–−0.01]	0.3394	0.3413
TBR_{mean}^{own} ratio	14	12	3.17 [2.61–3.43]	3.38 [2.95–4.36]	+0.52 [−0.77–1.33]	0.3258	2.91 [2.49–4.20]	2.53 [2.35–3.46]	−0.43 [−0.83–0.34]	0.1763	0.1897
TBR_{peak}^{DOPA}	14	12	3.19 [2.67–3.77]	2.88 [2.57–3.03]	−0.33 [−0.93–0.20]	0.2166	3.11 [2.17–3.57]	2.75 [2.37–3.96]	+0.09 [−0.67–0.40]	0.9697	0.3961
TBR_{peak}^{FDG}	14	12	1.00 [0.87–1.20]	0.88 [0.84–0.98]	−0.06 [−0.24–0.01]	0.1040	0.98 [0.90–1.09]	1.03 [0.93–1.14]	−0.02 [−0.09–0.17]	0.6221	0.1427
TBR_{peak}^{own} ratio	14	12	3.06 [2.87–3.41]	3.17 [2.82–3.58]	−0.08 [−0.42–0.72]	0.7148	2.91 [2.36–4.06]	2.62 [2.41–3.47]	+0.09 [−0.58–0.21]	0.6772	0.4875
