# Supplementary material for: Paired [18F]FDOPA and [18F]FDG PET Shows Complementary Prognostic Associations at Recurrence in IDH-Wildtype Glioblastoma
Source: Cancers (Basel). 2026 Oct 15;18(20):3333. doi: 10.3390/cancers18203333 (PMC13702550; doi:10.3390/cancers18203333)
Supplement: Supplementary file 1 [file cancers-18-03333-s001.zip › Table_S2_revised_FDR_final.pdf]

Table S2. Univariate Kaplan–Meier and Cox analyses for all PET imaging metrics by spatial domain at baseline (OS1) and recurrence (OS2).

Patients dichotomized at cohort median for Kaplan–Meier analysis. OS1: origin = PET1 (n = 36, events = 28). OS2: origin = PET2_latest (n = 55, events = 44). Univariate Cox HR expressed per +1 SD (no covariate adjustment; volumes log-transformed then standardized). TBRmean = MeanSUV/reference; TBRpeak = PeakSUV/reference (CM for FDG, CM_WM for DOPA). Ratio = DOPA/FDG. Bold red: $p < 0.05$. Benjamini–Hochberg q-values were calculated separately across the 42 continuous Cox screening tests at OS1 and OS2.

Metric	OS1 (baseline PET1)				OS2 (recurrence PET2)			
	n	Median	Log-rank p	Univariate HR (per +1 SD); p; BH q	n	Median	Log-rank p	Univariate HR (per +1 SD); p; BH q
**MRI-based**								
WT volume (mL)	36	59.0	0.7287	0.95 (0.67–1.36); 0.7910; q = 0.852	55	88.3	0.1992	1.25 (0.92–1.70); 0.1504; q = 0.421
**Union (A∪B)**								
MTV^{union} (mL)	36	20.1	0.4125	1.19 (0.83–1.68); 0.3427; q = 0.648	55	24.1	0.0821	**1.36 (1.00–1.84); 0.0487**; q = 0.332
SUV_{mean}^{DOPA}	36	1.787	0.4950	1.06 (0.74–1.52); 0.7550; q = 0.845	55	1.488	0.2660	1.32 (0.97–1.80); 0.0791; q = 0.332
SUV_{mean}^{FDG}	36	4.980	0.2583	0.97 (0.67–1.40); 0.8592; q = 0.880	55	4.102	0.3458	0.87 (0.61–1.23); 0.4214; q = 0.668
SUV_{mean}^{union} ratio	36	0.374	0.7991	1.10 (0.75–1.63); 0.6190; q = 0.743	55	0.381	**0.0043**	**1.62 (1.17–2.24); 0.0035**; q = 0.049
SUV_{peak}^{DOPA}	36	2.511	0.7067	1.17 (0.79–1.74); 0.4337; q = 0.648	55	2.371	0.9056	1.38 (0.98–1.95); 0.0669; q = 0.332
SUV_{peak}^{FDG}	36	6.867	0.7604	0.85 (0.59–1.23); 0.3944; q = 0.648	55	6.183	0.4525	0.92 (0.68–1.23); 0.5680; q = 0.745
SUV_{peak}^{union} ratio	36	0.393	0.5782	1.39 (0.92–2.09); 0.1156; q = 0.536	55	0.377	0.1357	1.23 (0.93–1.62); 0.1501; q = 0.421
TBR_{mean}^{DOPA}	36	2.101	0.4752	1.10 (0.76–1.59); 0.6059; q = 0.743	55	1.675	**0.0121**	1.19 (0.86–1.63); 0.2942; q = 0.639
TBR_{mean}^{FDG}	36	0.690	0.2173	1.00 (0.69–1.44); 0.9851; q = 0.985	55	0.610	0.4029	1.15 (0.84–1.56); 0.3947; q = 0.668
TBR_{mean}^{union} ratio	36	2.838	0.3583	1.17 (0.76–1.82); 0.4726; q = 0.662	55	2.682	0.5418	1.03 (0.78–1.36); 0.8257; q = 0.882
TBR_{peak}^{DOPA}	36	3.236	0.7332	1.16 (0.79–1.70); 0.4473; q = 0.648	55	2.616	0.6666	1.15 (0.83–1.58); 0.3986; q = 0.668
TBR_{peak}^{FDG}	36	1.001	0.4525	0.80 (0.55–1.17); 0.2541; q = 0.648	55	0.913	0.2282	1.29 (0.95–1.75); 0.0992; q = 0.347
TBR_{peak}^{union} ratio	36	2.945	0.1723	1.49 (1.00–2.22); 0.0525; q = 0.536	55	2.684	0.2593	0.97 (0.72–1.31); 0.8606; q = 0.882
**Intersection (A∩B)**								
MTV^{inter} (mL)	36	15.6	0.6028	1.39 (0.91–2.13); 0.1278; q = 0.536	55	12.3	**0.0110**	**1.72 (1.22–2.43); 0.0020**; q = 0.049
SUV_{mean}^{DOPA}	36	2.001	0.7102	1.04 (0.71–1.51); 0.8479; q = 0.880	55	1.796	0.6887	1.04 (0.75–1.45); 0.8062; q = 0.882
SUV_{mean}^{FDG}	36	5.550	0.8929	0.88 (0.60–1.28); 0.4915; q = 0.666	55	4.532	0.3020	0.82 (0.59–1.14); 0.2327; q = 0.611
SUV_{mean}^{inter} ratio	36	0.385	0.7685	1.30 (0.86–1.95); 0.2151; q = 0.648	55	0.421	0.1500	1.14 (0.86–1.51); 0.3661; q = 0.668
SUV_{peak}^{DOPA}	36	2.511	0.7067	1.21 (0.82–1.79); 0.3348; q = 0.648	55	2.371	0.9056	1.37 (0.97–1.95); 0.0741; q = 0.332
SUV_{peak}^{FDG}	36	6.531	0.5586	0.89 (0.61–1.29); 0.5267; q = 0.691	55	6.050	0.8198	1.01 (0.75–1.37); 0.9355; q = 0.935
SUV_{peak}^{inter} ratio	36	0.393	0.5782	1.43 (0.96–2.12); 0.0790; q = 0.536	55	0.416	0.6244	1.18 (0.89–1.57); 0.2556; q = 0.621
TBR_{mean}^{DOPA}	36	2.581	0.1272	1.06 (0.74–1.50); 0.7642; q = 0.845	55	1.979	0.5561	0.89 (0.64–1.24); 0.5051; q = 0.737
TBR_{mean}^{FDG}	36	0.758	0.6734	0.84 (0.57–1.23); 0.3617; q = 0.648	55	0.706	0.8466	1.04 (0.77–1.41); 0.7872; q = 0.882
TBR_{mean}^{inter} ratio	36	3.028	0.2162	1.36 (0.90–2.07); 0.1484; q = 0.536	55	3.065	0.2145	0.87 (0.65–1.15); 0.3252; q = 0.650
TBR_{peak}^{DOPA}	36	3.189	0.3381	1.25 (0.86–1.82); 0.2470; q = 0.648	55	2.616	0.6666	1.14 (0.83–1.57); 0.4292; q = 0.668
TBR_{peak}^{FDG}	36	0.923	0.7771	0.84 (0.59–1.20); 0.3412; q = 0.648	55	0.894	0.1908	**1.34 (1.00–1.80); 0.0487**; q = 0.332
TBR_{peak}^{inter} ratio	36	2.945	**0.0487**	**1.62 (1.07–2.45); 0.0239**; q = 0.536	55	2.798	0.2735	0.91 (0.68–1.22); 0.5262; q = 0.737
**Own masks**								
MTV^{DOPA} (mL)	36	18.6	0.4125	1.15 (0.82–1.62); 0.4164; q = 0.648	55	17.7	**0.0026**	**1.66 (1.19–2.32); 0.0027**; q = 0.049
MTV^{FDG} (mL)	36	17.4	0.6028	1.55 (0.99–2.41); 0.0541; q = 0.536	55	19.7	0.0628	1.35 (1.00–1.82); 0.0532; q = 0.332
MTV^{own} ratio	36	1.0	**0.0483**	0.71 (0.48–1.04); 0.0754; q = 0.536	55	0.9	0.7122	1.03 (0.82–1.31); 0.7754; q = 0.882
SUV_{mean}^{DOPA}	36	1.892	0.7151	1.09 (0.75–1.59); 0.6504; q = 0.759	55	1.726	0.9575	1.06 (0.75–1.48); 0.7531; q = 0.882
SUV_{mean}^{FDG}	36	5.360	0.8929	0.84 (0.58–1.23); 0.3751; q = 0.648	55	4.330	0.5723	0.85 (0.62–1.16); 0.3042; q = 0.639
SUV_{mean}^{own} ratio	36	0.383	0.1596	1.38 (0.92–2.07); 0.1224; q = 0.536	55	0.422	0.1362	1.07 (0.83–1.38); 0.6064; q = 0.772
SUV_{peak}^{DOPA}	36	2.511	0.7067	1.17 (0.79–1.74); 0.4337; q = 0.648	55	2.371	0.9056	1.38 (0.98–1.95); 0.0669; q = 0.332
SUV_{peak}^{FDG}	36	6.867	0.7604	0.85 (0.59–1.23); 0.3944; q = 0.648	55	6.183	0.4525	0.92 (0.68–1.23); 0.5680; q = 0.745
SUV_{peak}^{own} ratio	36	0.393	0.5782	1.39 (0.92–2.09); 0.1156; q = 0.536	55	0.377	0.1357	1.23 (0.93–1.62); 0.1501; q = 0.421
TBR_{mean}^{DOPA}	36	2.478	0.1272	1.11 (0.78–1.57); 0.5649; q = 0.719	55	1.888	0.4164	0.90 (0.65–1.24); 0.5200; q = 0.737
TBR_{mean}^{FDG}	36	0.733	0.6734	0.80 (0.54–1.18); 0.2666; q = 0.648	55	0.663	0.5275	1.07 (0.79–1.44); 0.6703; q = 0.828
TBR_{mean}^{own} ratio	36	3.121	**0.0213**	1.28 (0.91–1.78); 0.1532; q = 0.536	55	2.899	0.2145	0.86 (0.65–1.13); 0.2662; q = 0.621
TBR_{peak}^{DOPA}	36	3.236	0.7332	1.16 (0.79–1.70); 0.4473; q = 0.648	55	2.616	0.6666	1.15 (0.83–1.58); 0.3986; q = 0.668
TBR_{peak}^{FDG}	36	1.001	0.4525	0.80 (0.55–1.17); 0.2541; q = 0.648	55	0.913	0.2282	1.29 (0.95–1.75); 0.0992; q = 0.347
TBR_{peak}^{own} ratio	36	2.945	0.1723	1.49 (1.00–2.22); 0.0525; q = 0.536	55	2.684	0.2593	0.97 (0.72–1.31); 0.8606; q = 0.882
