# Supplementary material for: Paired [18F]FDOPA and [18F]FDG PET Shows Complementary Prognostic Associations at Recurrence in IDH-Wildtype Glioblastoma
Source: Cancers (Basel). 2026 Oct 15;18(20):3333. doi: 10.3390/cancers18203333 (PMC13702550; doi:10.3390/cancers18203333)
Supplement: Supplementary file 1 [file cancers-18-03333-s001.zip › Table_S3.pdf]

Table S3. Multivariable Cox proportional hazards models for overall survival at baseline (OS1) and recurrence (OS2): univariately significant PET metrics.

Univariately significant PET metrics (Table S2) tested individually in multivariable Cox models adjusted for age, MGMT, initial surgery, and WHO performance status. HR per +1 SD; volumes log-transformed and standardized; Efron ties. References: CM (FDG), CM_WM (DOPA). Ratio = DOPA/FDG. Bold red: $p < 0.05$.

Panel A. OS1 (time origin: baseline PET1; n = 36, events = 28) — 3 metrics.

Metric	HR (95% CI); p
**Intersection (A∩B)**	
TBR_{peak}^{inter} ratio	0.97 (0.63–1.49); 0.8874
**Own masks**	
MTV^{own} ratio	1.08 (0.69–1.68); 0.7295
TBR_{mean}^{own} ratio	0.82 (0.58–1.18); 0.2873

Panel B. OS2 (time origin: recurrence PET2_latest; n = 55, events = 44) — 6 metrics.

Metric	HR (95% CI); p
**Union (A∪B)**	
MTV^{union} (mL)	1.26 (0.90–1.78); 0.1804
SUV_{mean}^{union} ratio	**1.50 (1.08–2.09); 0.0168**
TBR_{mean}^{DOPA}	1.21 (0.86–1.70); 0.2695
**Intersection (A∩B)**	
MTV^{inter} (mL)	**1.63 (1.10–2.42); 0.0154**
TBR_{peak}^{FDG}	1.14 (0.77–1.69); 0.5218
**Own masks**	
MTV^{DOPA} (mL)	**1.58 (1.08–2.31); 0.0191**
