# Supplementary material for: Paired [18F]FDOPA and [18F]FDG PET Shows Complementary Prognostic Associations at Recurrence in IDH-Wildtype Glioblastoma
Source: Cancers (Basel). 2026 Oct 15;18(20):3333. doi: 10.3390/cancers18203333 (PMC13702550; doi:10.3390/cancers18203333)
Supplement: Supplementary file 1 [file cancers-18-03333-s001.zip › Table_S4.pdf]

Table S4. Stepwise derivation of IR-MTV: which combination of PET biomarkers adds prognostic value without redundancy?

OS2 (n = 55, events = 44). Multivariable Cox models adjusted for age, MGMT, surgery, and WHO performance status. C-index: Harrell's apparent concordance; C.632: bootstrap-corrected concordance (B = 100). VIF (variance inflation factor) shown for biomarker pairs (Step 3): VIF > 5 indicates redundancy. Bold red row: retained combination defining IR-MTV^{DOPA} = Ratio SUVmean (union) × log(MTV DOPA).

Step	Component / combination	VIF (max)	C-index	C.632	ΔC vs base
Step 1 — Clinical base model (reference)					
1	Base = Age + MGMT + surgery + WHO performance status	—	0.668	0.610	—
Step 2 — Each biomarker added individually (univariable gain)					
2a	Base + SUV_{mean}^{union} ratio	—	0.701	0.650	+0.033
2b	Base + MTV^{DOPA}	—	0.685	0.640	+0.017
2c	Base + MTV^{inter}	—	0.689	0.648	+0.022
Step 3 — Pairs of biomarkers (screen for synergy and redundancy)					
**3a**	**Base + SUV_{mean}^{union} ratio + MTV^{DOPA} ← IR-MTV^{DOPA} components**	**1.39**	**0.738**	**0.671**	**+0.070**
3b	Base + SUV_{mean}^{union} ratio + MTV^{inter} (alternative)	1.46	0.735	0.673	+0.068
3c	Base + MTV^{DOPA} + MTV^{inter} (redundant)	**19.16**	0.689	0.643	+0.022
